# Supplementary material for: TIAR marks nuclear G2/M transition granules and restricts CDK1 activity under replication stress
Source: EMBO Rep. 2018 Dec 11;20(1):e46224. doi: 10.15252/embr.201846224 (PMC6322364; doi:10.15252/embr.201846224)
Supplement: Supplementary file 1 — Appendix [file EMBR-20-e46224-s001.pdf]

Appendix

TIAR marks nuclear G2/M transition granules and restricts CDK1 activity under replication stress

Vanesa Lafarga, Hsu-Min Sung, Katharina Haneke, Lea Roessig, Anne-Laure Pauleau, Marius Bruer, Sara Rodriguez-Acebes, Andres Lopez-Contreras, Oliver J. Gruss, Sylvia Erhardt, Juan Mendez, Oscar Fernandez-Capetillo and Georg Stoecklin

Table of contents

**Appendix Figure S1**	S-phase and DNA fiber analysis	page 2
**Appendix Figure S2**	Chromatin bridge and metaphase analysis	page 3
**Appendix Figure S3**	M-phase, S-phase and DNA fiber analysis after replication stress	page 4
**Appendix Figure S4**	Cell cycle analysis and multinucleated cells upon TIAR kd	page 5
**Appendix Figure S5**	CDK1 inhibition induces formation of GMGs	page 6
**Appendix Figure S6**	GMG formation in different cell lines	page 7
**Appendix Figure S7**	Characterization of GMGs	page 8
**Appendix Figure S8**	GMG formation is TIAR-independent	page 9
**Appendix Figure S9**	Analysis of mitotic cells and CDK1 activation status	page 10

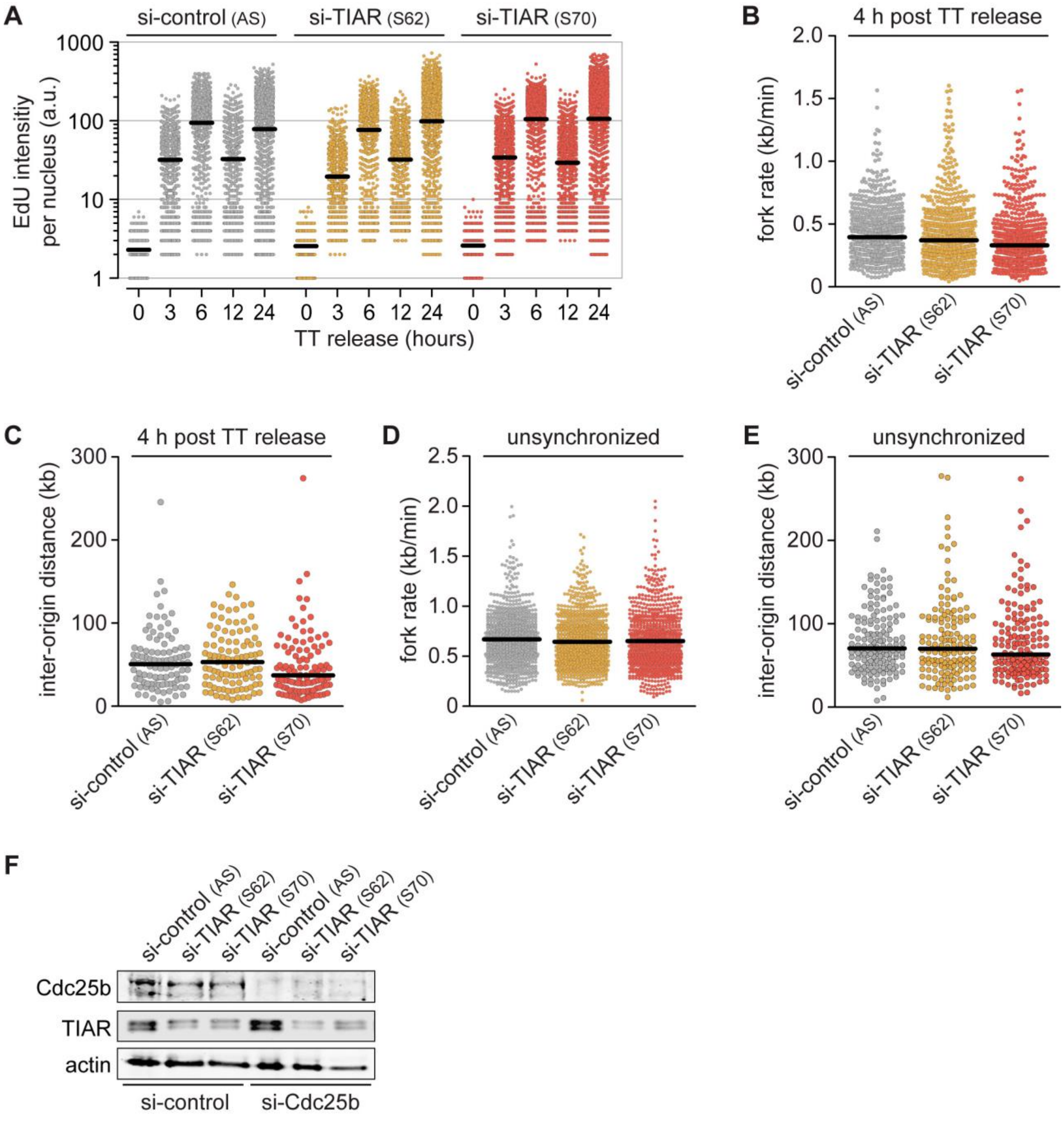

Appendix Figure S1. S-phase and DNA fiber analysis.

A HeLa cells were transfected with control or TIAR siRNAs for 48 hours and synchronized by TT block. After release from the block, cells were labeled with EdU 30 minutes prior to fixation and EdU-incorporation was quantified by HTM (n = 3, 1000 cells examined per experiment and condition).

B HeLa cells were transfected with control or TIAR siRNAs for 48 hours and synchronized by TT block. Fork rate was measured by DNA fiber analysis 4 hours after release. Shown is the average of n = 3 independent experiments.

C DNA fiber analysis was carried out as in (B) to measure the inter-origin distance.

D HeLa cells were transfected with control or TIAR siRNAs, and fork rate was measured by DNA fiber analysis. Shown is the average of n = 3 independent experiments.

E DNA fiber analysis was carried out as in (D) to measure the inter-origin distance.

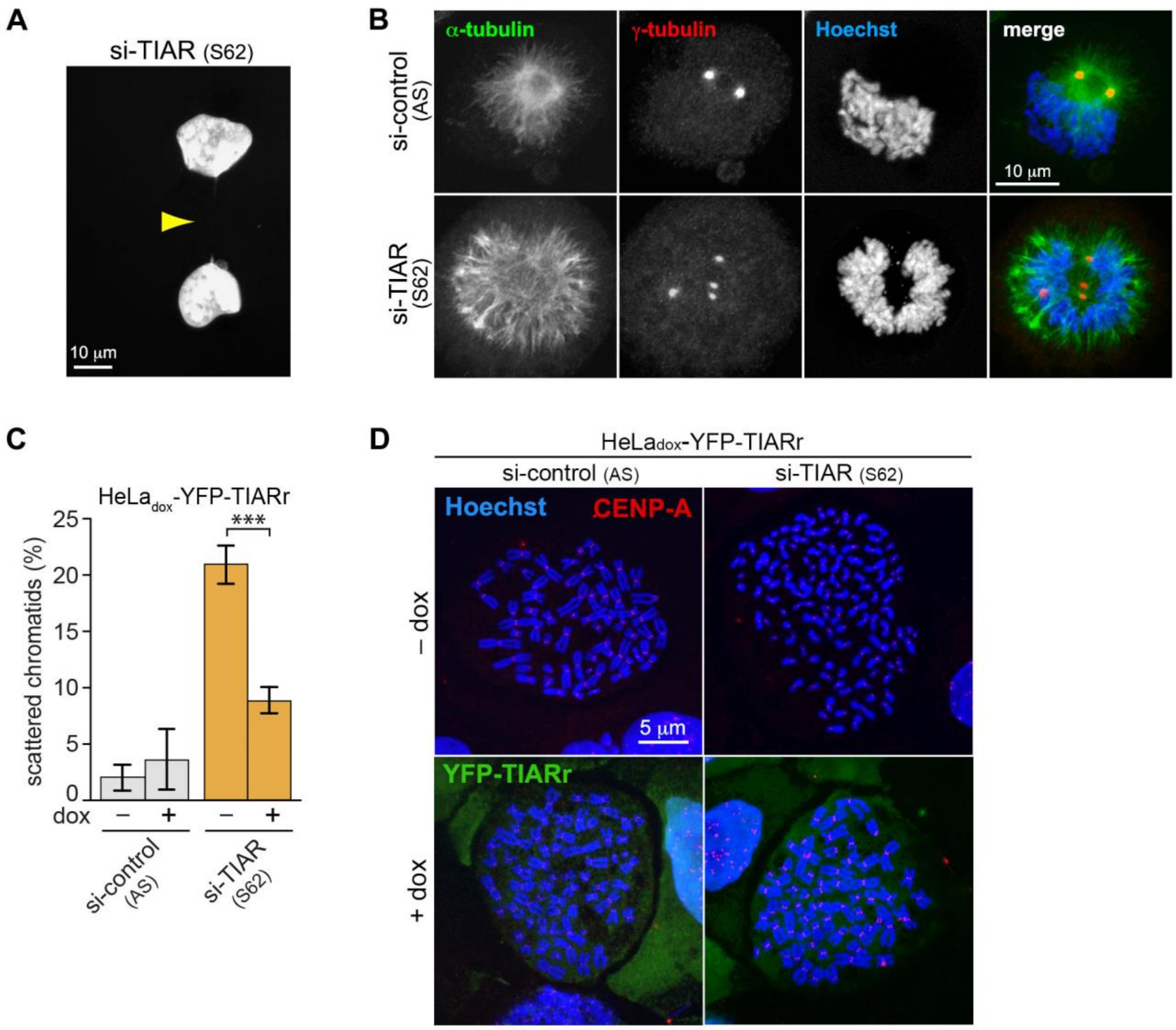

Appendix Figure S2. Chromatin bridge and metaphase analysis.

A HeLa cells were transfected with TIAR siRNA for 72 hours and prepared for fluorescence microscopy after staining with Hoechst. The yellow arrowhead points to a chromatin bridge.

B HeLa cells were transfected with control or TIAR siRNAs for 72 hours, and mitotic cells were analyzed by IF microscopy after staining with anti-α-tubulin antibody, anti-γ-tubulin antibody and Hoechst.

C $HeLa_{dox}$-YFP-TIARr cells were transfected with control or TIAR siRNAs and cultured in the absence or presence of doxycycline. Following preparation of metaphase spreads, the frequency of metaphase spreads with scattered chromatids was quantified (mean ± SD, n = 3).

D Metaphase spreads were prepared as in (C), and chromosomes were stained with Hoechst (blue) and anti-CENP-A antibody (red). The green signal corresponds to YFP-TIARr.

Data information: In (C), statistical significance was determined by unpaired Student's t-test; ***, $p < 0.001$.

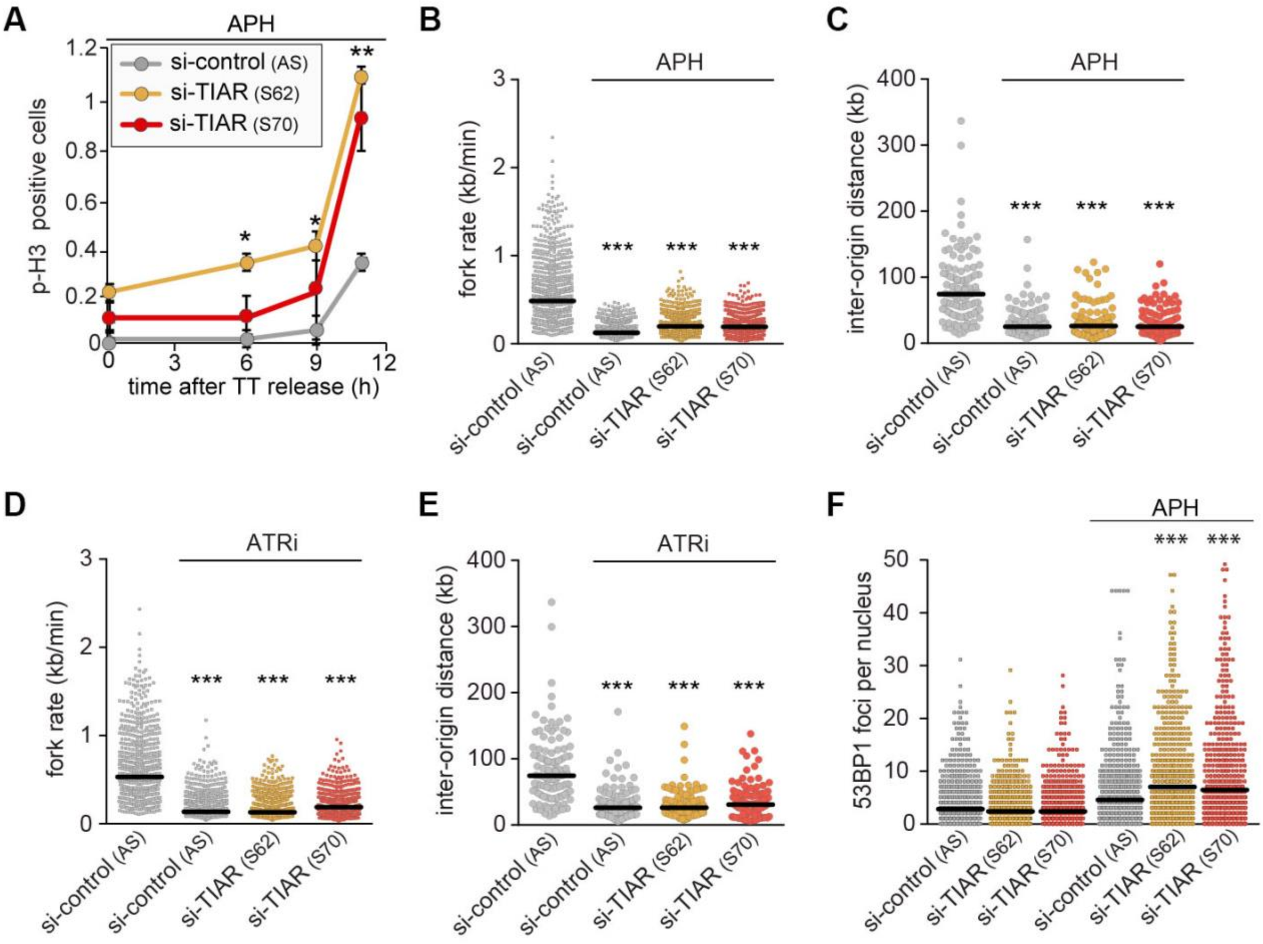

Appendix Figure S3. M-phase, S-phase and DNA fiber analysis after replication stress.

A HeLa-H2B/tub cells were transfected with control or TIAR siRNAs for 48 hours and synchronized by TT block. After release from the block in presence of APH (0.4 µM), mitotic cells were counted by fluorescence microscopy (mean ± SD, n = 3).

B HeLa cells were transfected with control or TIAR siRNAs for 72 hours and then treated for 1 hour with APH (1 µM). Fork rate was measured by DNA fiber analysis (data combined from 2 independent experiments.

C DNA fiber analysis was carried out as in (B) to measure the inter-origin distance.

D HeLa cells were transfected with control or TIAR siRNAs for 72 hours and then treated for 1 hour with ATRi (2.5 µM). Fork rate was measured by DNA fiber analysis (data combined from 2 independent experiments.

E DNA fiber analysis was carried out as in (D) to measure the inter-origin distance.

F HeLa cells were transfected with control or TIAR siRNAs for 48 hours prior to treatment with 0.4 µM APH. Cells were fixed and stained with anti-53BP1 antibody. The number of 53BP1 foci was quantified by HTM (n = 3, 2000 cells examined per experiment and condition).

Data information: In (A), statistical significance was determined by unpaired Student's t-test. In (B–F), statistical significance was determined by Wilcoxon rank-sum test; **, p < 0.01; ***, p < 0.001.

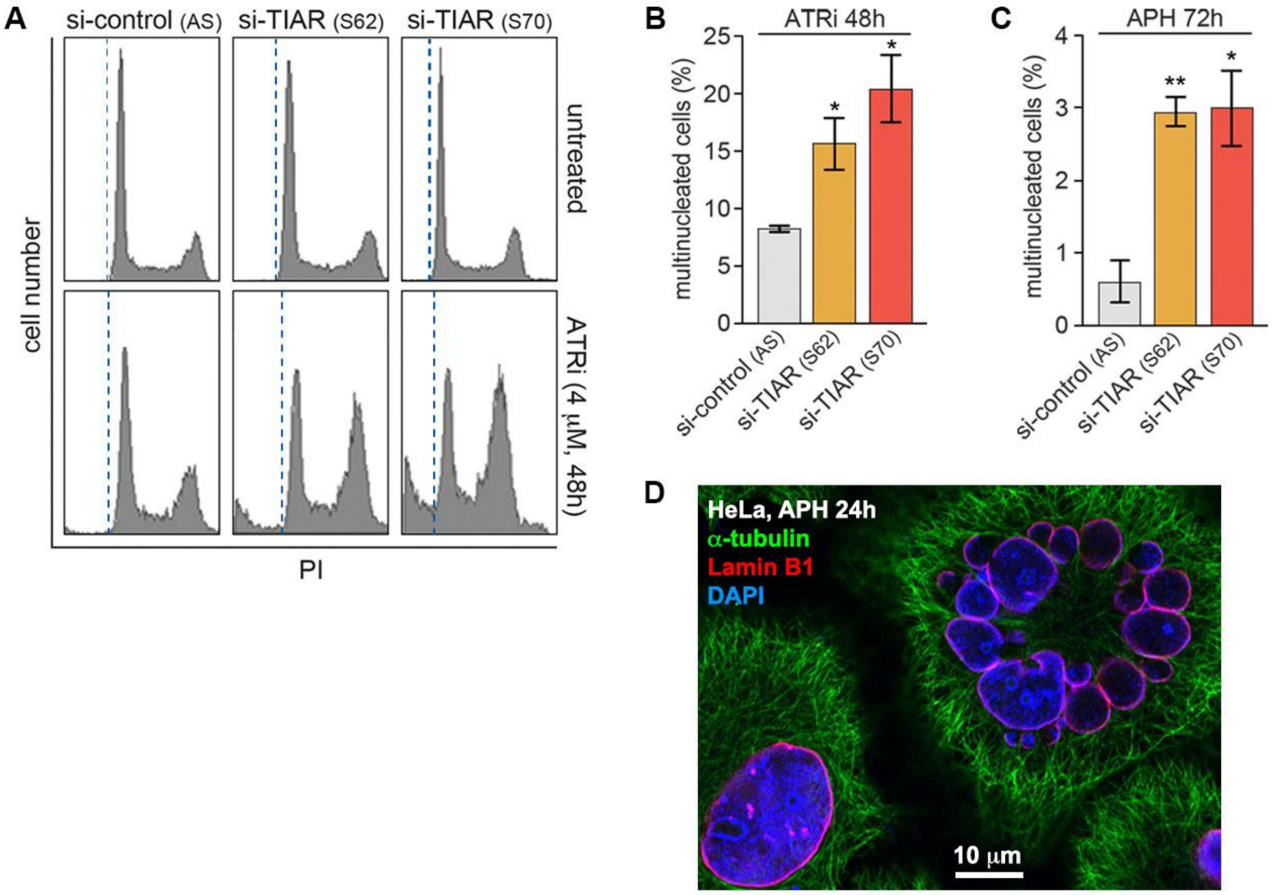

Appendix Figure S4. Cell cycle analysis and multinucleated cells upon TIAR kd.

A HeLa cells transfected with control or TIAR siRNAs were treated with 4 µM ATRi for 48 hours. For cell cycle profile analysis, cells were fixed, stained with propidium iodide and analyzed by flow cytometry.

B HeLa cells transfected with control or TIAR siRNAs were treated with 4 µM of ATRi for 48 hours, fixed and stained with anti-α-tubulin and anti-Lamin B1 antibodies. The frequency of multinucleated cells was determined by IF microscopy (mean ± SD, n = 3 independent experiments, approximately 500 cells were counted per condition and experiment).

C The frequency of multinucleated cells was determined as in (B) upon treatment of siRNA transfected HeLa cells with 0.4 µM APH for 72 hours (mean ± SD, n = 3).

D Image obtained by IF microscopy from the analysis in (C). The micrograph shows a multinucleated giant cell after kd of TIAR, indicative of mitotic catastrophe.

Data information: In (B, C), statistical significance was determined by unpaired Student's t-test; *, $p < 0.05$; **, $p < 0.01$.

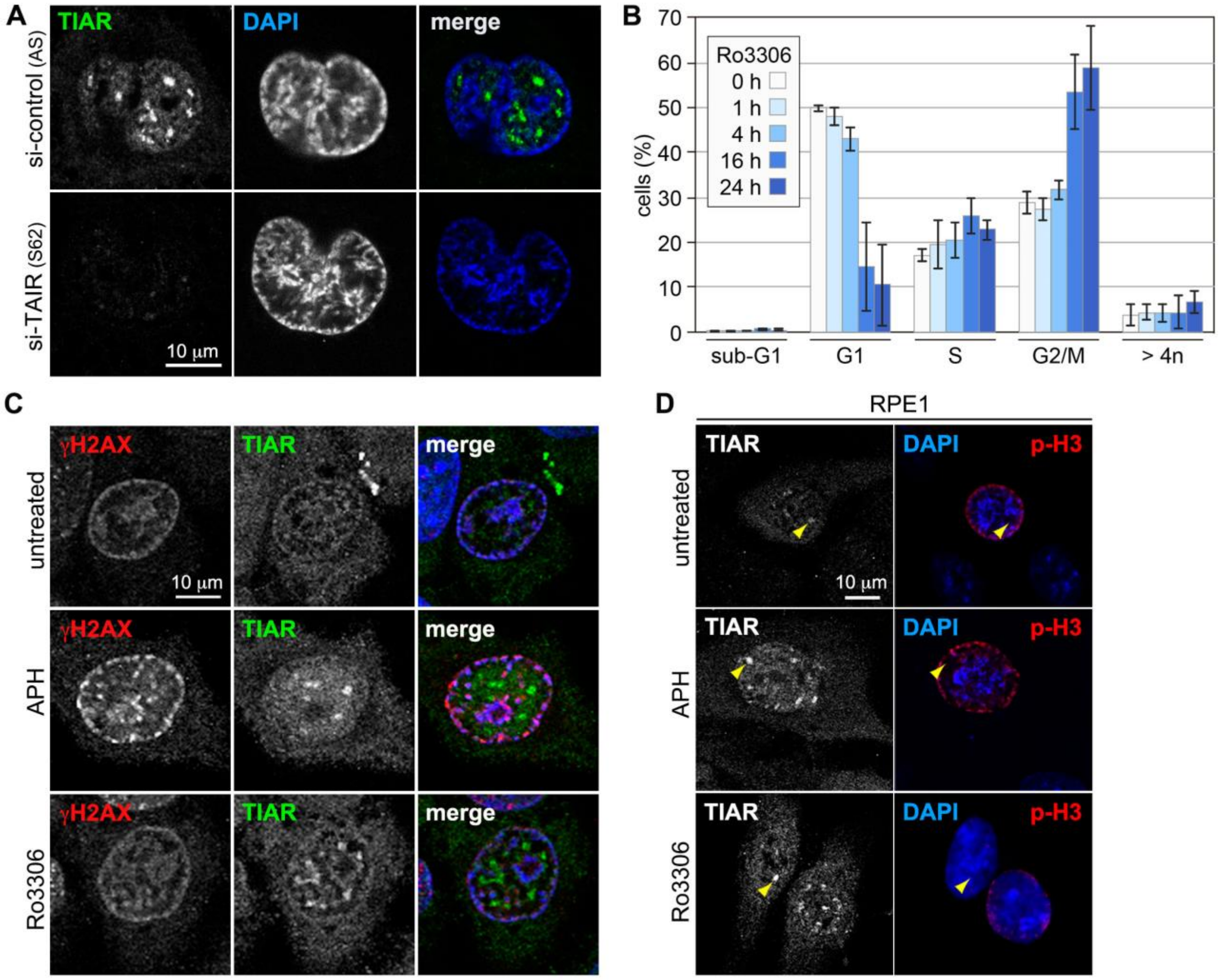

Appendix Figure S5. CDK1 inhibition induces formation of GMGs.

A HeLa cells transfected with control or TIAR siRNAs for 72 hours were treated with 0.4 µM APH for 16 hours and fixed with methanol. IF microscopy was performed after staining with anti-TIAR antibody and DAPI; images were taken with identical exposure settings.

B Hela cells were treated with Ro3306 (10 µM) and fixed at the indicated times. Cell cycle distribution was quantified by flow cytometry following propidium iodide staining (mean ± SD, n = 3).

C HeLa cell were treated for 16 hours with APH (0,4 µM) or Ro3306 (9 µM) prior to fixation with methanol and staining with anti-TIAR and anti-γH2AX antibodies.

D RPE1 cells were treated for 24 hours with APH (0,4 µM) or Ro3360 (10 µM), fixed with methanol and analyzed by IF microscopy after staining with anti-p(S10)-H3 and anti-TIAR antibodies. Yellow arrows mark GMGs.

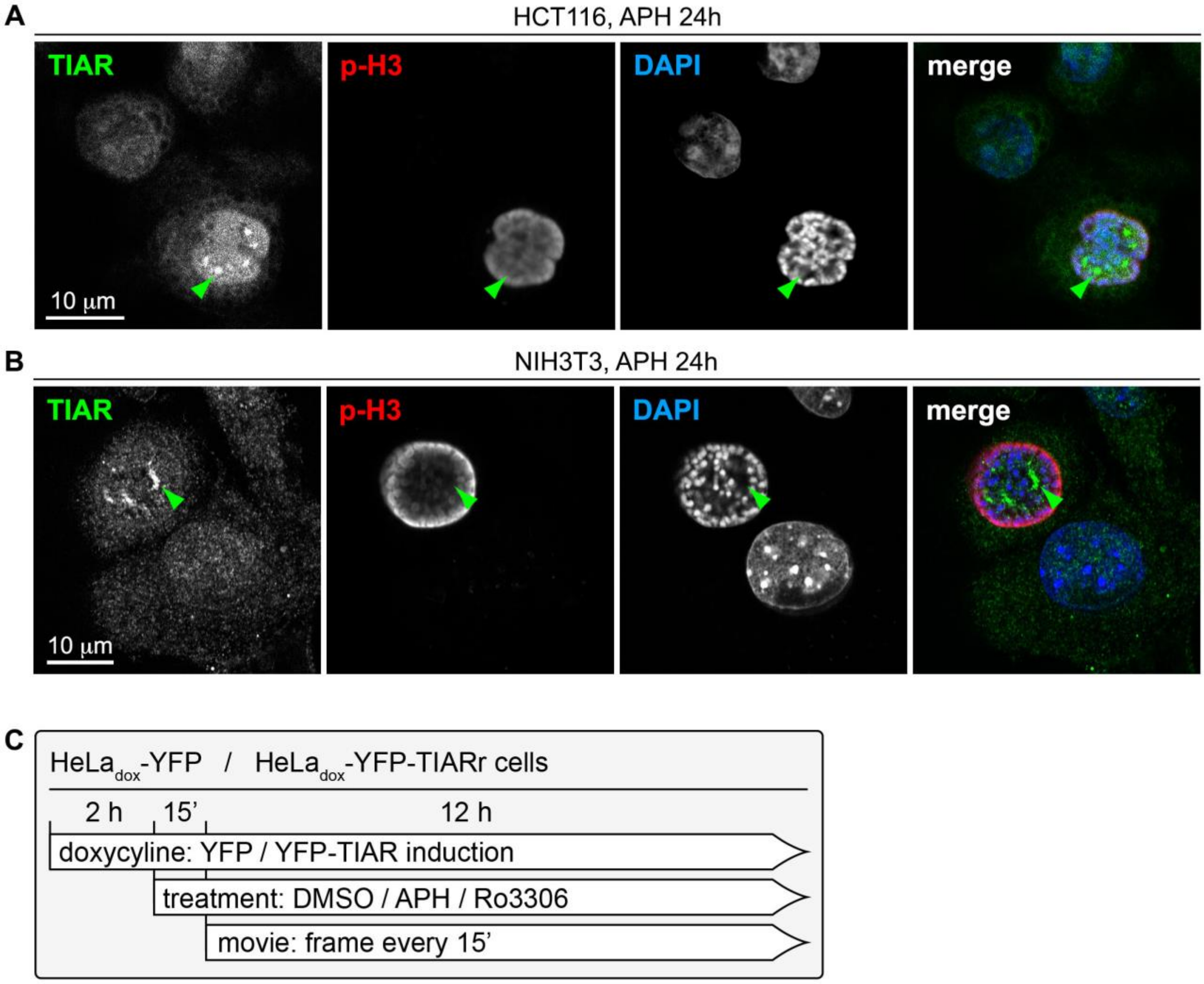

Appendix Figure S6. GMG formation in different cell lines.

A HCT116 cells were treated with APH (0.4 µM) for 24 hours, and fixed with methanol. IF microscopy was performed after staining with anti-p(S10)-H3 antibody, anti-TIAR antibody and DAPI. Green arrows mark GMGs.

B NIH3T3 cells were treated with APH (0.4 µM) for 24 hours, and IF microscopy was carried out as in (A).

C Scheme of experimental design for Movies EV1–4.

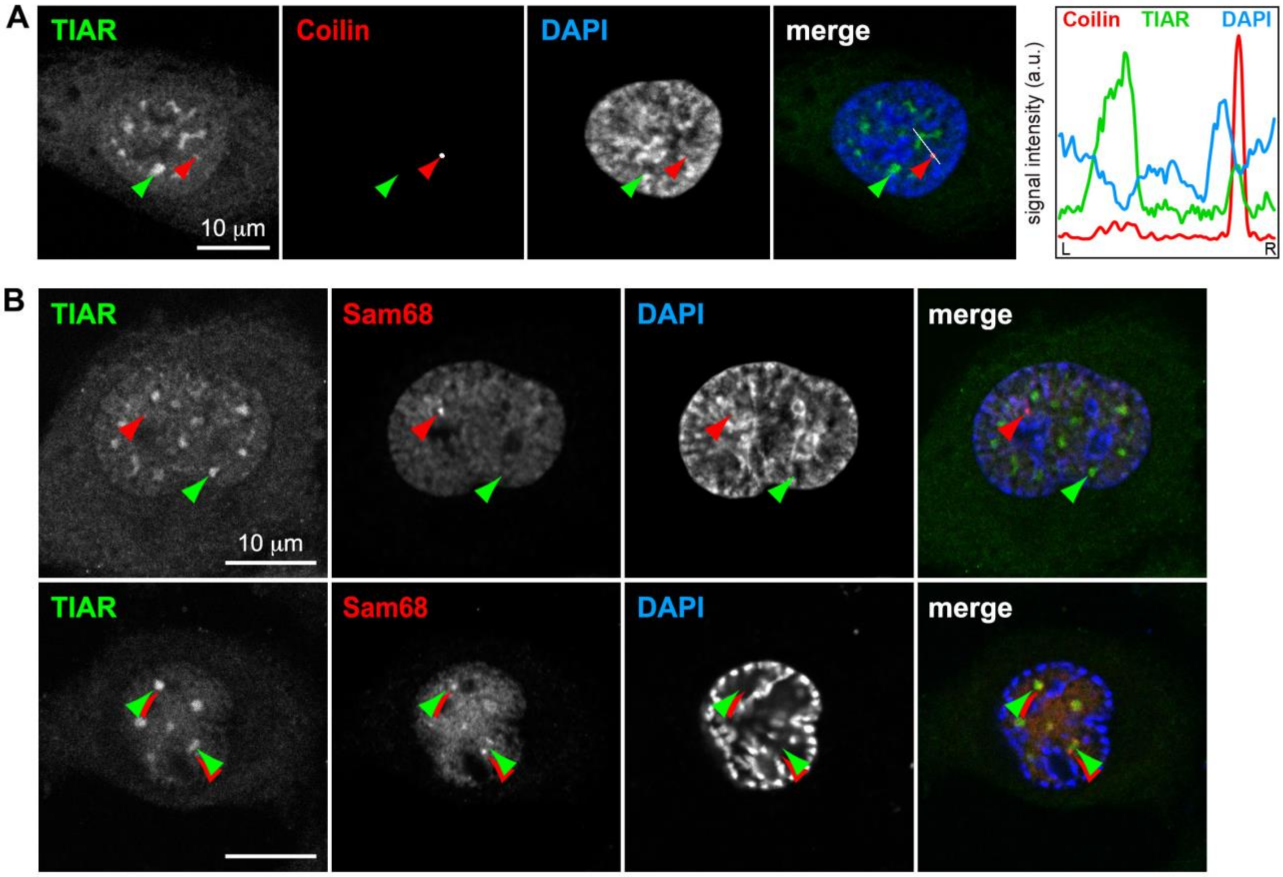

Appendix Figure S7. Characterization of GMGs.

A HeLa cells were treated with 0.4 µM APH for 24 hours, fixed with methanol and processed for IF microscopy after staining with DAPI, anti-TIAR antibody in combination with anti-Coilin antibody. Green arrows mark GMGs, red arrows mark nuclear foci that are distinct from GMGs.

B IF microscopy was carried out as in (A) in combination with anti-Sam68 antibody.

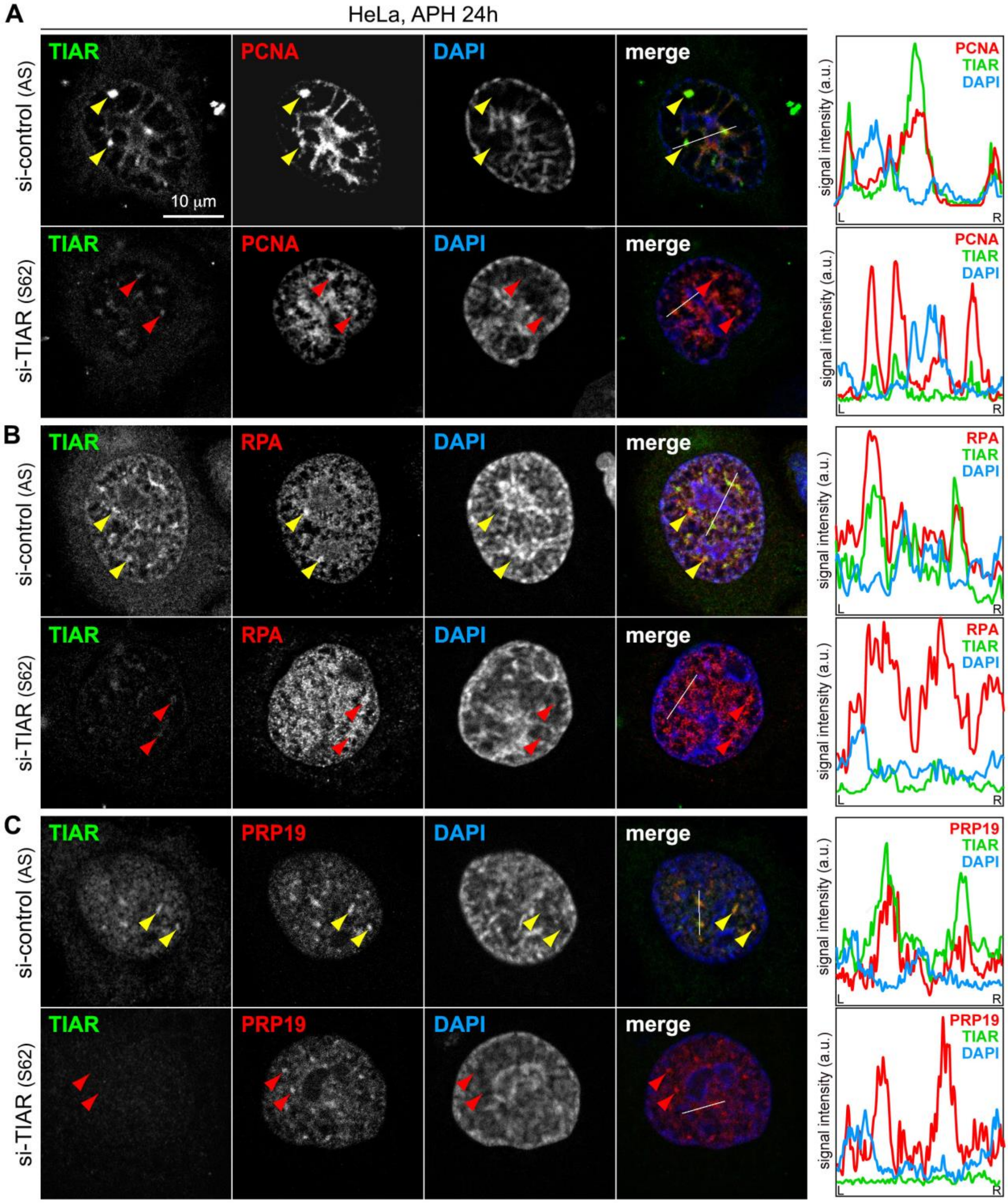

Appendix Figure S8. GMG formation is TIAR-independent.

A HeLa cells transfected with control or TIAR siRNAs for 48 hours were treated for 16 hours with 0.4 µM APH, fixed and processed for IF microscopy. Cells were stained with anti-TIAR antibody and DAPI in combination with anti-PCNA antibody.

B IF microscopy was carried out as in (A) in combination with anti-RPA antibody.

C IF microscopy was carried out as in (A) in combination with anti-PRP19 antibody.

Data information: Intensity profiles along the white line in the merged image are depicted on the right side.

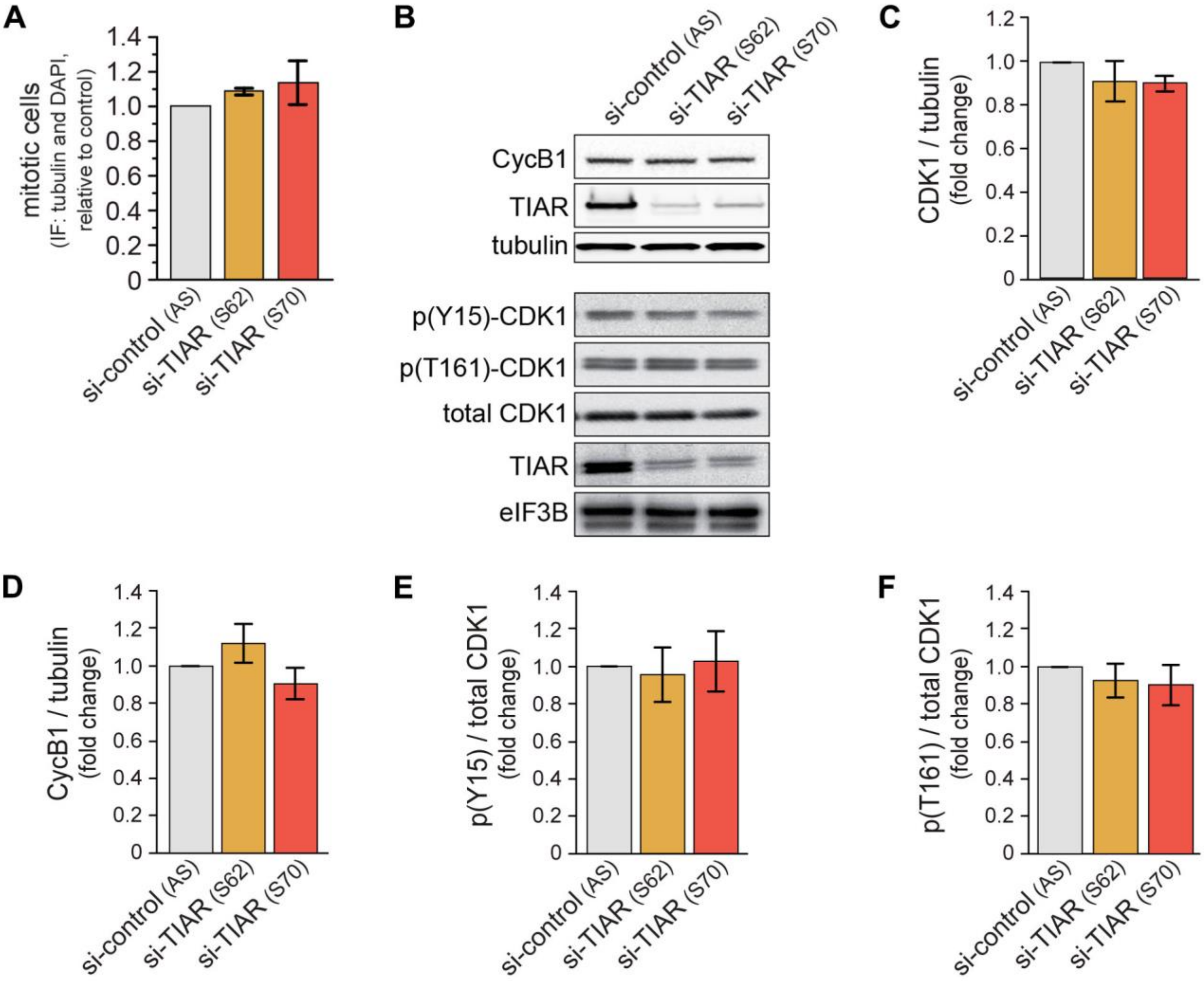

Appendix Figure S9. Analysis of mitotic cells and CDK1 activation status.

A HeLa cells were transfected with control or TIAR siRNAs for 72 hours, fixed and processed for IF microscopy after staining with mouse anti-tubulin antibody and DAPI. The average number of mitotic cells (from prophase to telophase) was determined by eye from n = 3 independent experiments, 200 cells were analyzed per experiment (mean ± SD).

B HeLa cells were transfected with control or TIAR siRNAs for 72 hours before measuring expression levels of CyclinB1 (CycB1), p(Y15)-CDK1 and p(T161)-CDK1 by western blot analysis.

C Quantification of total CDK1 levels normalized to tubulin based on western blot analysis (mean ± SD, n = 4).

D Quantification of CyclinB1 levels normalized to tubulin based on western blot analysis (mean ± SD, n = 4).

E Quantification of p(Y15)-CDK1 levels normalized to total CDK1 based on western blot analysis (mean ± SD, n = 4).

F Quantification of p(T161)-CDK1 levels normalized to total CDK1 based on western blot analysis (mean ± SD, n = 4).
