# Supplementary material for: TIAR marks nuclear G2/M transition granules and restricts CDK1 activity under replication stress
Source: EMBO Rep. 2018 Dec 11;20(1):e46224. doi: 10.15252/embr.201846224 (PMC6322364; doi:10.15252/embr.201846224)
Supplement: Supplementary file 2 — Expanded View Figures PDF [file EMBR-20-e46224-s002.pdf]

Expanded View Figures

Figure EV1. CDK1 inhibition antagonizes loss of TIAR.

A HeLa cells were transfected with control or TIAR siRNAs and, 72 h later, treated with (5 μM) ATRi alone or together with (5 μM) Ro3306 for 12 h. Chromatin-bound RPA intensity was quantified by HTM (n = 3; 1,000 cells examined per experiment and condition).

B, C HeLa cells were transfected with control or TIAR siRNAs, alone or together with Cdc25B siRNA. After 48 h, cells were treated with ATRi (4 μM) for 24 h and pan-nuclear γH2AX (B) or RPA-bound chromatin (C) were quantified by HTM (n = 3; 1,000 cells examined per experiment and condition).

D Pan-nuclear γH2AX signals were quantified by HTM in siRNA-transfected RPE1 cells 48 h after treatment with (5 μM) ATRi (n = 3; 1,000 cells examined per experiment and condition).

E, F HeLa cells were transfected with control or TIAR siRNAs for 48 h and synchronized by TT block. After release from the block in the presence of ATRi (5 μM), cells were fixed at the indicated timepoints and (E) pan-nuclear γH2AX levels were quantified by HTM. (F) Cells were labeled with EdU 30 min prior to fixation, and EdU incorporation was quantified by HTM. n = 3; 1,000 cells examined per experiment and condition.

Data information: In (A–D), statistical significance was determined by Wilcoxon rank-sum test; *$P < 0.05$; **$P < 0.01$; ***$P < 0.001$, ****$P < 0.0001$.

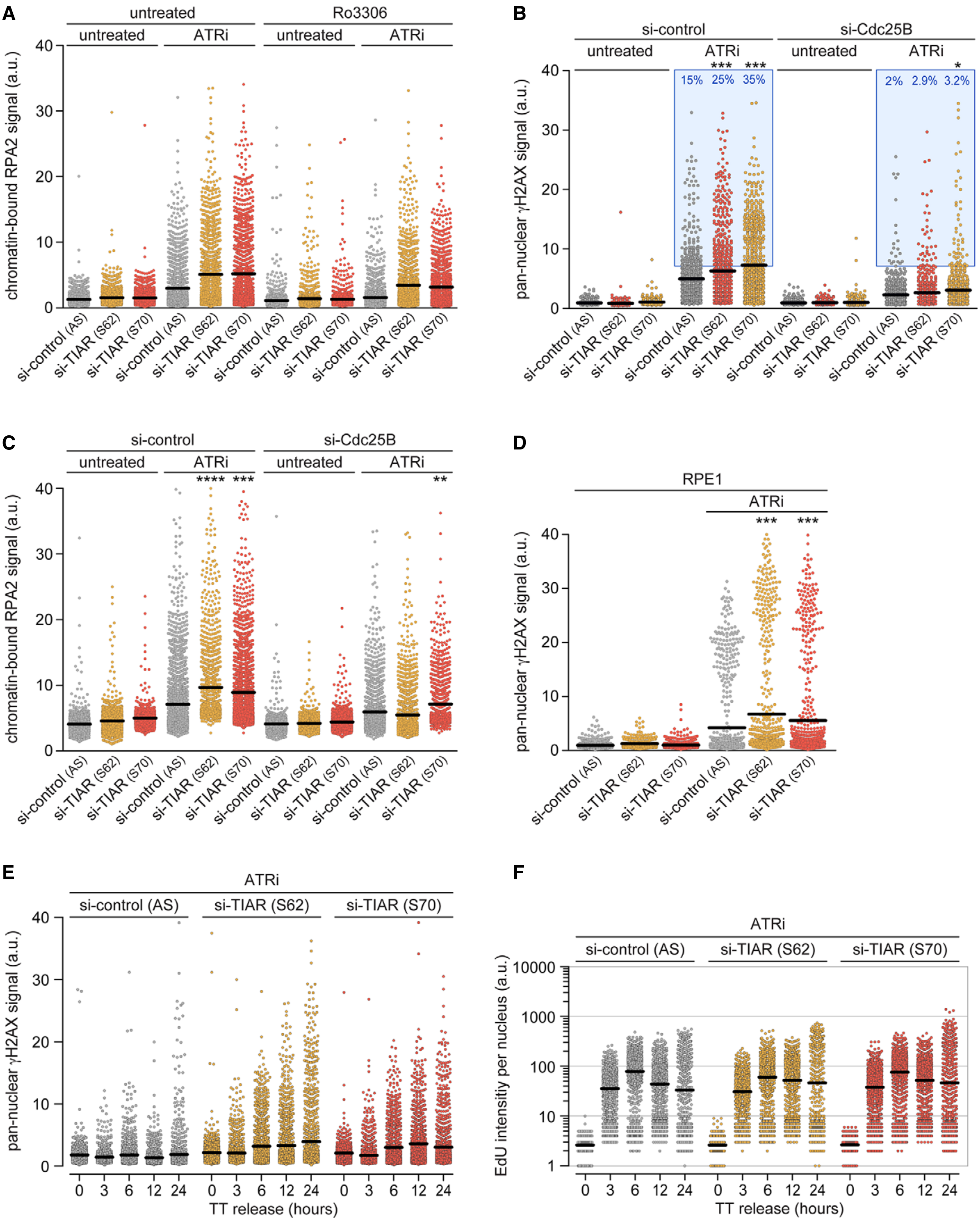

Figure EV1.

© 2018 The Authors

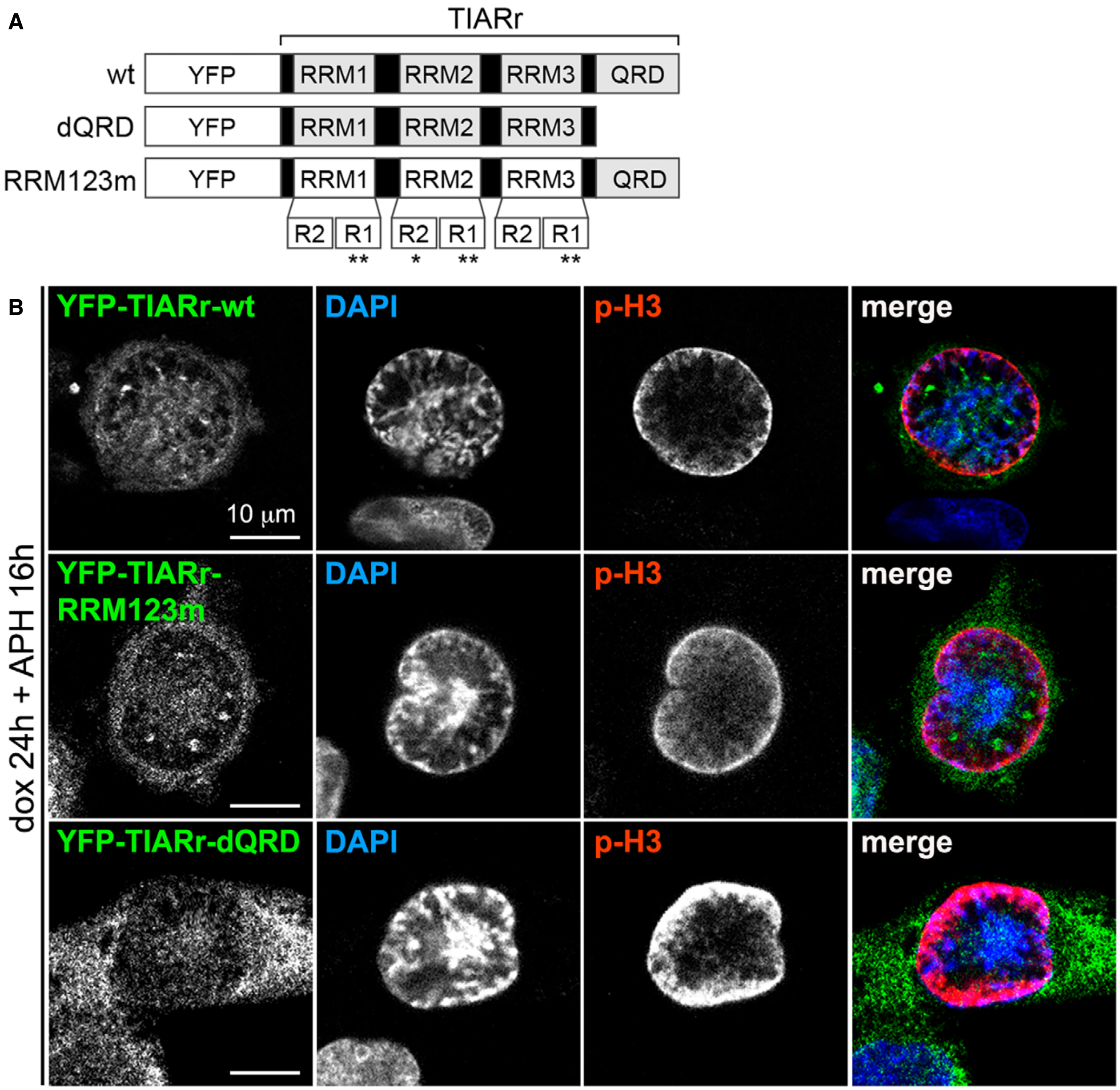

Figure EV2. QRD is required to localize TIAR in GMGs.

A Scheme of TIAR mutants. All TIARr plasmids contain a silent point mutation at amino acid V244 and R245, which renders the corresponding mRNA resistant to siRNA S62. In TIARr-dQRD, the C-terminus of TIAR (amino acids 284–375) was deleted by insertion of a thymidine residue before N284, thereby introducing a stop codon. TIARr-RRM123m has the following point mutations: Y50A, F52A, F100A, Y140A, F142A, Y242A, and Y244A, as indicated by asterisks.

B HeLa$_{dox}$-YFP-TIARr, HeLa$_{dox}$-YFP-TIARr-RRM123m, and HeLa$_{dox}$-YFP-TIARr-dQRD cells were transfected with control or TIAR siRNAs, and cultured in the absence or presence of 200 μM doxycycline for 24 h. Cells were treated with 0.4 μM APH for an additional 16 h, fixed with methanol, and analyzed by IF microscopy after staining with anti-p(S10)-H3 antibody and DAPI.

© 2018 The Authors

Figure EV3. TIA1 kd does not lead to premature mitotic entry or chromosomal defects.

A HeLa cells were treated for 24 h with 0.4 μM APH, fixed with methanol, and analyzed by IF microscopy after staining with anti-p(S10)-H3 antibody, anti-TIA1 antibody, and DAPI.

B Quantification of late G2/prophase cells with visible GMGs upon staining of TIA1 or TIAR by IF microscopy (mean ± SD, n = 3 independent experiments, 40 cells were analyzed per experiment and condition).

C Western blot analysis was carried out to monitor expression of p(S10)-H3, total H3, TIA1, and TIAR in control, TIA1-, and TIAR-depleted cells.

D, E HeLa cells were transfected with control, TIA1, or TIAR siRNAs 48 h prior to treatment with 0.4 μM APH. (D) Cells were collected at regular time intervals, and expression of p(S10)-H3, total H3, TIA1, and TIAR was monitored by Western blot analysis; eIF3B serves as loading control. (E) Cells were fixed at regular time intervals, and p(S10)-H3-positive cells were quantified by flow cytometry (mean values ± SEM, n = 3).

F, G HeLa cells were transfected with control, TIA1, or TIAR siRNAs for 4 days and subjected to colcemid for 2 h prior to preparation of metaphase spreads. The frequency of cells with (F) chromosomal breaks or (G) scattered chromatids was quantified from metaphase spreads (mean ± SD, n = 3 independent experiments, approximately 30 metaphase spreads were assessed per experiment and condition).

Data information: In (B, F and G), statistical significance was determined by unpaired Student's t-test; ***$P < 0.001$.

 © 2018 The Authors

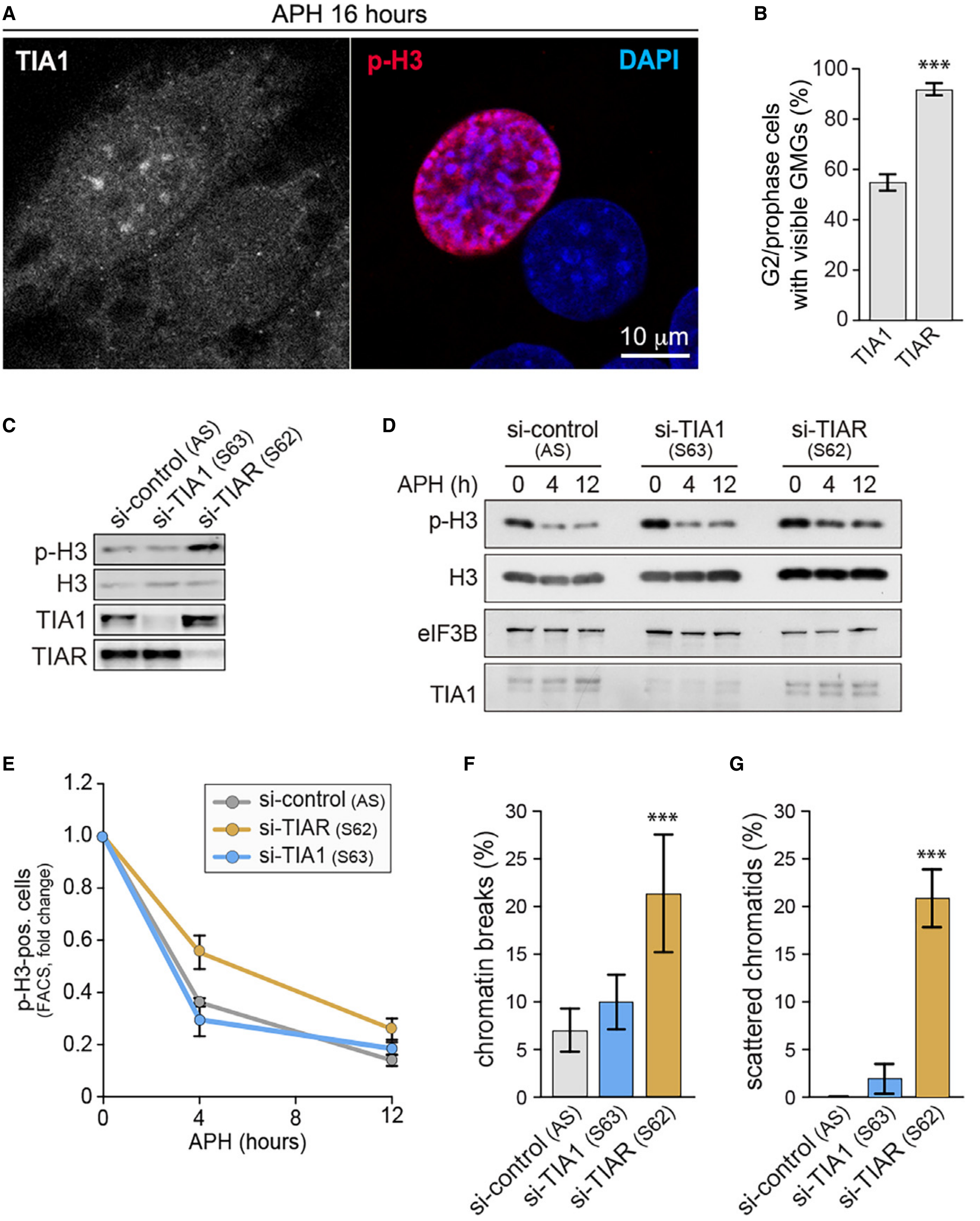
A
APH 16 hours
TIA1
p-H3
DAPI
10 μm
B
G2/prophase cells with visible GMGs (%)
***
TIA1
TIAR
C
si-control (AS)
si-TIA1 (S63)
si-TIAR (S62)
p-H3
H3
TIA1
TIAR
D
si-control (AS)
si-TIA1 (S63)
si-TIAR (S62)
APH (h)
0 4 12
p-H3
H3
eIF3B
TIA1
E
si-control (AS)
si-TIAR (S62)
si-TIA1 (S63)
p-H3-pos. cells (FACS, fold change)
APH (hours)
F
chromatin breaks (%)
***
si-control (AS)
si-TIA1 (S63)
si-TIAR (S62)
G
scattered chromatids (%)
***
si-control (AS)
si-TIA1 (S63)
si-TIAR (S62)

Figure EV3.

Figure EV4. Localization of TIAR with respect to different markers.

A Quantification of the percentage of GMGs containing PCNA, BRCA1, FANCD2, PRP19, Sm, HuR, pS2-RPOL2A, RPA1, RNase H1, 53BP1, γH2AX, and Rad51; 70 GMGs were analyzed per condition.

B HeLa cells were treated with 0.4 μM APH for 24 h, fixed with methanol, and processed for IF microscopy after staining with anti-TIAR antibody and DAPI in combination with anti-RPA1 antibody.

C IF microscopy was carried out as in (B) in combination with anti-RNase H1 antibody.

D HeLa cells were treated with 0.4 μM APH for 24 h and labeled with 10 μM 5-ethynyl-deoxyuridine (EdU) during the last 1 h of treatment to mark newly synthesized DNA. EdU was visualized using click chemistry, and IF microscopy was carried out after staining with anti-TIAR antibody and DAPI.

E IF microscopy was carried out as in (B) in combination with anti-53BP1 antibody.

F IF microscopy was carried out as in (B) in combination with anti-γH2AX antibody.

G IF microscopy was carried out as in (B) in combination with anti-Rad51 antibody.

Data information: In (B–G), intensity profiles along the white line in the merged image are depicted on the right side. Yellow and green arrows mark GMGs, and red arrows mark nuclear foci that are distinct from GMGs.

 © 2018 The Authors

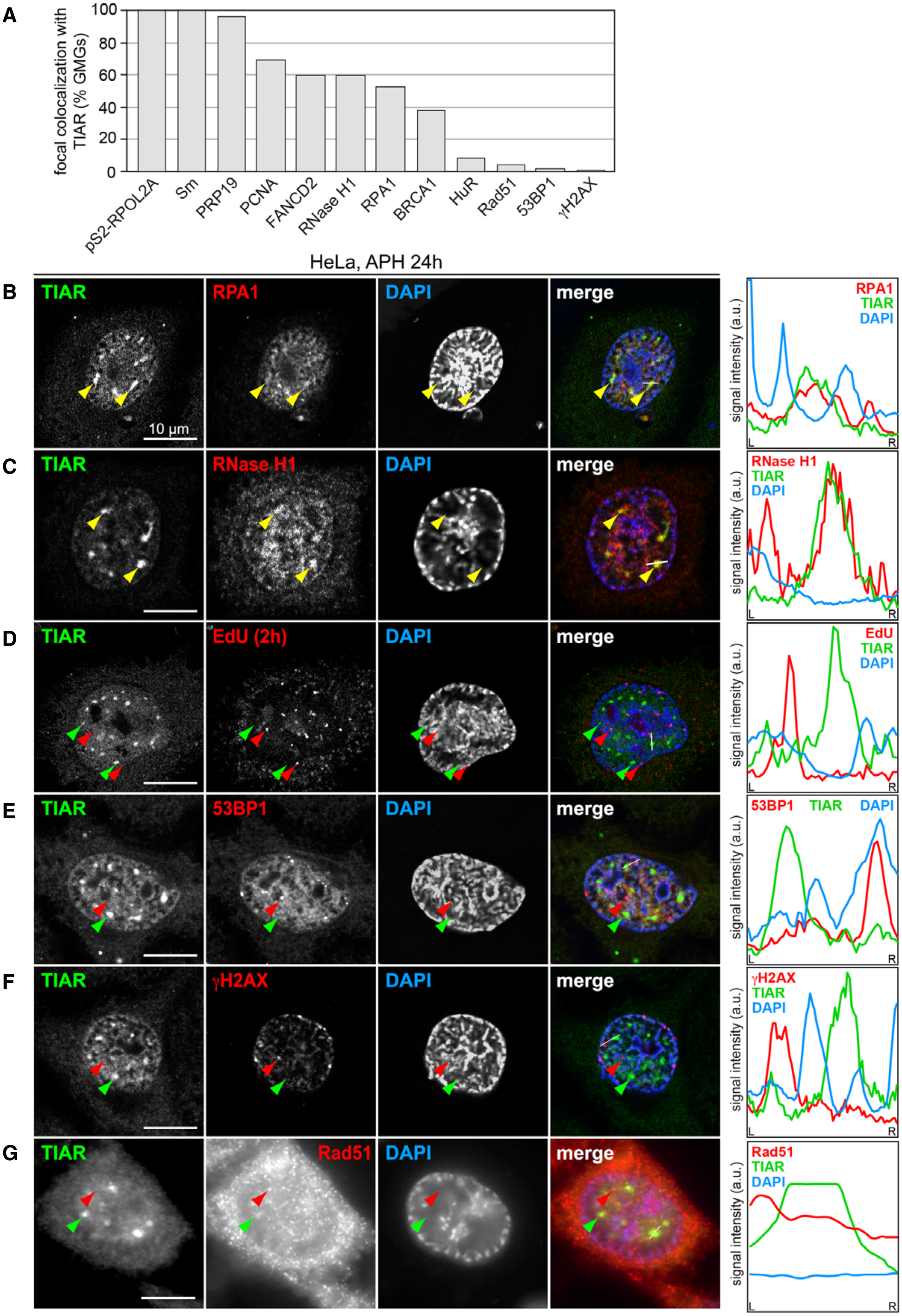
A
focal colocalization with TIAR (% GMGs)
100
80
60
40
20
0
pS2-RPOL2A
Sm
PRP19
PCNA
FANCD2
RNase H1
RPA1
BRCA1
HuR
Rad51
53BP1
γH2AX
HeLa, APH 24h
B
TIAR
RPA1
DAPI
merge
10 μm
C
TIAR
RNase H1
DAPI
merge
D
TIAR
EdU (2h)
DAPI
merge
E
TIAR
53BP1
DAPI
merge
F
TIAR
γH2AX
DAPI
merge
G
TIAR
Rad51
DAPI
merge
signal intensity (a.u.)
L
R

Figure EV4.

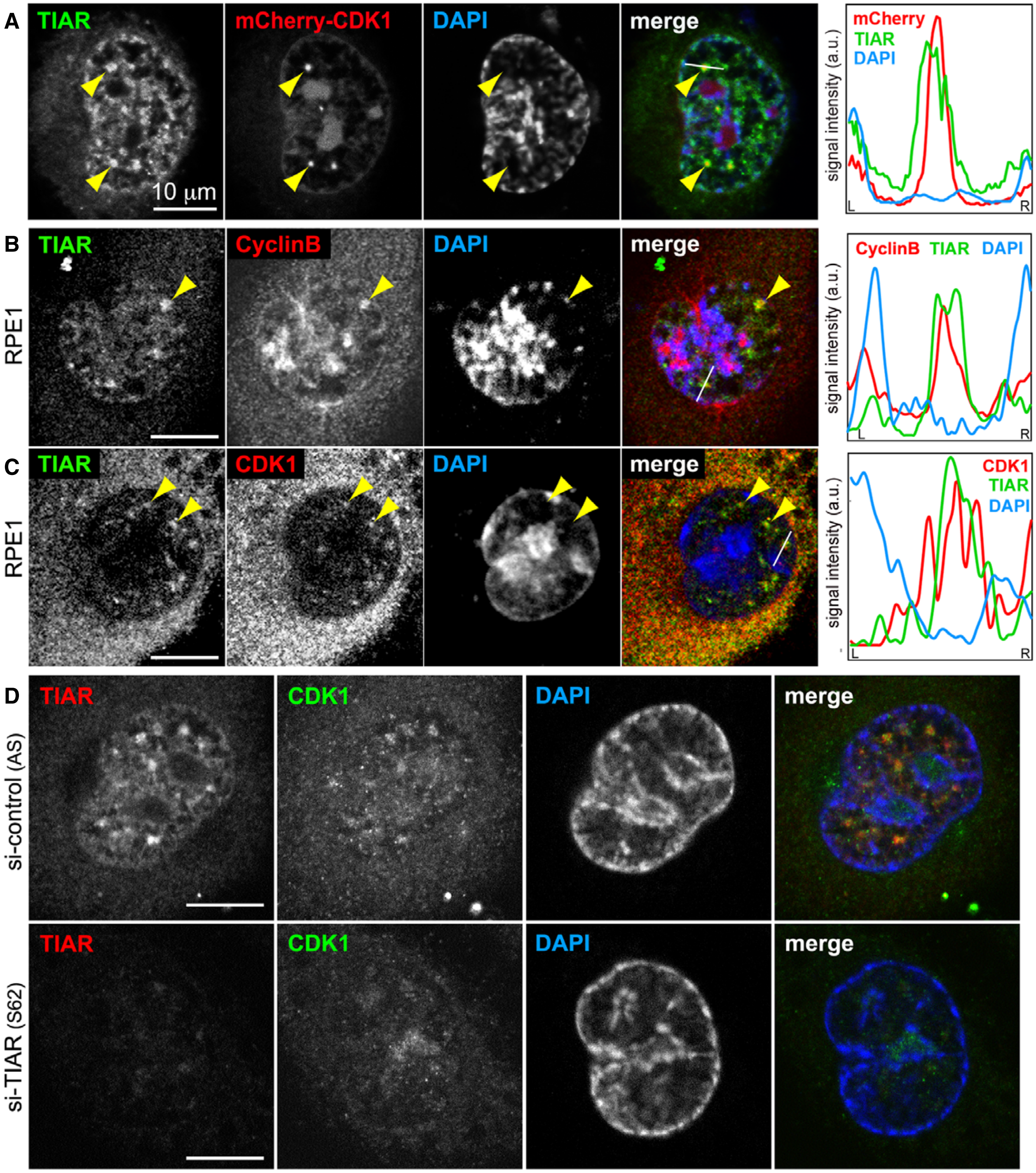

Figure EV5. Retention of CDK1 by TIAR in GMGs.

A HeLa cells were transfected with mCherry-CDK1 and 24 later treated with 0.4 μM APH for 16 h. Cells were then fixed with methanol and processed for IF microscopy after staining with anti-TIAR antibody and DAPI.

B RPE1 cells were treated with 0.4 μM APH for 24 h, fixed, and processed for IF microscopy after staining with anti-TIAR antibody and DAPI in combination with anti-Cyclin B antibody.

C IF microscopy was carried out as in (B) in combination with anti-CDK1 antibody.

D HeLa cells transfected with control or TIAR siRNAs were treated with APH 24 h prior to fixation. Cells were stained with goat anti-TIAR antibody, mouse anti-CDK1 antibody, and DAPI for IF microscopy.

Data information: In (A–C), intensity profiles along the white line in the merged image are presented on the right side; yellow arrows mark GMGs.

 © 2018 The Authors
